# Cardiovascular Rehabilitation for transient ischaemic Attack and Mild Stroke: the CRAMS effectiveness-implementation hybrid study protocol

**DOI:** 10.1186/s12913-022-08797-3

**Published:** 2022-11-22

**Authors:** Nicole Freene, Hannah Wallett, Allyson Flynn, Elisabeth Preston, Shahla Cowans, Christian Lueck, Theophile Niyonsenga, Itismita Mohanty, Rachel Davey

**Affiliations:** 1 Physiotherapy, Faculty of Health, University of Canberra, Bruce, ACT Australia; 2 Health Research Institute, University of Canberra, Bruce, ACT Australia; 3 Neurology, Canberra Health Services, Garran, ACT Australia

**Keywords:** Stroke, Transient ischeamic attack, Secondary prevention, Cardiac rehabilitation, Effectiveness, Implementation, Qualitative

## Abstract

**Background:**

Internationally, stroke and cardiac rehabilitation clinicians agree that current cardiac rehabilitation models are a suitable secondary prevention program for people following a transient ischaemic attack (TIA) or mild stroke. There is strong evidence for exercise-based cardiac rehabilitation in people with heart disease, however, the evidence for cardiac rehabilitation post-TIA or stroke is limited. Here we will explore the effectiveness and implementation of an integrated (TIA, mild stroke, heart disease) traditional exercise-based cardiovascular rehabilitation (CVR) program for people with TIA or mild stroke over 6-months.

**Methods:**

This type 1 effectiveness-implementation hybrid study will use a 2-arm single-centre assessor-blind randomised controlled trial design, recruiting 140 participants. Adults who have had a TIA or mild stroke in the last 12-months will be recruited by health professionals from hospital and primary healthcare services. Participants will be assessed and randomly allocated (1:1) to the 6-week CVR program or the usual care 6-month wait-list control group. Distance completed in the 6-min walk test will be the primary effectiveness outcome, with outcomes collected at baseline, 6-weeks (complete CVR) and 6-months in both groups. Other effectiveness outcome measures include unplanned cardiovascular disease-related emergency department and hospital admissions, daily minutes of accelerometer moderate-to-vigorous physical activity, body mass index, waist circumference, blood pressure, quality of life, anxiety and depression. Implementation outcomes will be assessed using the Reach, Effectiveness, Adoption, Implementation, Maintenance (RE-AIM) framework, including a cost-effectiveness analysis. Semi-structured interviews will be conducted with participants and CVR program health professionals, investigating the acceptability, value, and impact of the CVR program. Qualitative analyses will be guided by the Consolidated Framework for Implementation Research.

**Discussion:**

Few studies have assessed the effectiveness of cardiac rehabilitation for people with TIA and mild stroke, and no studies appear to have investigated the cost-effectiveness or implementation determinants of such programs. If successful, the CVR program will improve health outcomes and quality of life of people who have had a TIA or mild stroke, guiding future research, policy, and clinical practice, reducing the risk of repeat heart attacks and strokes for this population.

**Trial registration:**

Australian New Zealand Clinical Trials Registry (ANZCTR): ACTRN12621001586808, Registered 19 November 2021.

**Supplementary Information:**

The online version contains supplementary material available at 10.1186/s12913-022-08797-3.

## Background

In Australia and globally, cardiovascular disease (CVD) is the leading cause of death [1, 2]. CVD includes disorders of the heart and blood vessels such as coronary heart disease, ischaemic stroke and transient ischaemic attack (TIA) [3]. One in three CVD events are repeat events and these are more likely to be fatal and result in a higher burden of disease cost than initial events [4–6]. Recurrent stroke occurs in 11% of stroke survivors within one year, and 39% of stroke survivors within 10 years [7]. In addition, during the first 90 days following a TIA, individuals have a 15–20% risk of suffering a stroke [8]. Cardiac disease and recurrent stroke are the leading causes of mortality after stroke [9, 10]. In 2020, there were 445,000 stroke survivors living in Australia, and this is predicted to increase to an estimated 820,000 in 2050 [11]. The current international recommendation is that every person with CVD should be referred to a secondary prevention program, such as cardiac rehabilitation, to prevent recurrent CVD events, such as stroke and heart attacks [12–14]. However, evidence suggests that minimal secondary prevention services are provided post-stroke and TIA [15, 16].

There is strong evidence for exercise-based cardiac rehabilitation in people with heart disease, which can be defined as a comprehensive secondary prevention program which addresses modifiable risk factors for CVD using exercise and education [17]. Ischaemic stroke, TIA and coronary heart disease share many modifiable risk factors such as hypertension, smoking, physical inactivity, poor diet, obesity and excessive alcohol consumption [18]. Cardiac rehabilitation improves risk profiles, decreases hospital admissions and mortality, increases medication adherence and improves quality of life in those with heart disease [17]. It has been reported that a high proportion of stroke survivors have poor knowledge of stroke risk factors [19, 20] and control of cardiovascular health is suboptimal post stroke [21, 22]. Thus, cardiac rehabilitation may be a suitable option for secondary prevention for people with TIA and stroke [23, 24].

Previous research has found that despite the proposed suitability of cardiac rehabilitation, people with stroke or TIA are not routinely attending cardiac rehabilitation programs around the world [25, 26]. In Australia, a national survey of cardiac rehabilitation coordinators, conducted in 2018, found that less than 2% of the cardiac rehabilitation population were people with a diagnosis of stroke/TIA, and less than 6% of cardiac rehabilitation coordinators received referrals for this group of patients in the previous year [25]. However, over half of the cardiac rehabilitation coordinators thought that cardiac rehabilitation was appropriate for people with a primary diagnosis of TIA (60%) or mild stroke (54%). Transient symptoms, minimal disability from a mild stroke and similar risk factors for stroke and cardiac disease were common reasons reported for why mild stroke or TIA patients should be included in cardiac rehabilitation. Recently, the views of stroke health professionals in Australia were explored, finding that only 30% of stroke health professionals referred people with TIA and mild stroke to secondary prevention programs, despite over 90% of stroke health professionals believing cardiac rehabilitation to be an appropriate form of secondary prevention [27]. The top three barriers to cardiac rehabilitation for this population as perceived by the stroke health professionals were the name of the program, lack of referrals and lack of funding [27]. Both cardiac rehabilitation and stroke health professionals agreed that the similar exercise, education and supervision needs of people with cardiac disease and TIA or mild stroke were key enablers for cardiac rehabilitation inclusion [27]. Similar results have been found in surveys of cardiac rehabilitation and stroke health professionals in Canada, Switzerland and Ireland [23, 26, 28, 29].

The evidence for people with TIA or stroke attending cardiac rehabilitation is limited. One large randomised controlled trial has recently been reported, the PREVENT trial, finding modest effects on vascular risk factors for stroke following a 12-week exercise and education program for people with TIA or mild stroke [30]. Two small randomised controlled trials have also been identified in the literature, although it is unclear whether these studies are stroke only cardiac rehabilitation programs or integrated programs (TIA, stroke, heart disease) [31, 32]. Additionally, the inclusion criteria differ between studies. One study included participants > 1 year post stroke and any severity [31], while the other studies included people with TIA and mild stroke within one month [32] and three months [30] of diagnosis. There is some evidence of integrated cardiac rehabilitation programs, including participants with TIA, mild stroke and coronary heart disease, using pre-post study designs [33–35]. Despite this limited evidence, all studies found that the cardiac rehabilitation model was feasible in this population, improving fitness and reducing cardiovascular disease risk [30–35]. Utilising existing cardiac rehabilitation programs for people with TIA or mild stroke may positively impact health status without creating new programs, but more robust evidence is required to determine its impact on physical function, risk factors, quality of life, morbidity and mortality, including the feasibility of conducting integrated cardiac rehabilitation programs within health services and its cost-effectiveness [26]. With no consensus on comprehensive secondary prevention programs for people post-TIA or stroke at present, and existing cardiac rehabilitation programs being a potentially suitable option, our research questions are:

1. Is an integrated (TIA, mild stroke, heart disease) traditional exercise-based cardiac rehabilitation program effective in improving functional exercise capacity (6-min walk test distance) and quality of life, and reducing the risk of unplanned CVD-related hospital admissions, for people with TIA or mild stroke during the 6-months following commencement of the traditional cardiac rehabilitation program compared to usual care?
2. Can an integrated traditional exercise-based cardiac rehabilitation program be implemented in existing health services, considering implementation outcome measures such as acceptability, feasibility, adoption, fidelity, and cost?

## Methods

### Design

**Fig. 1:**
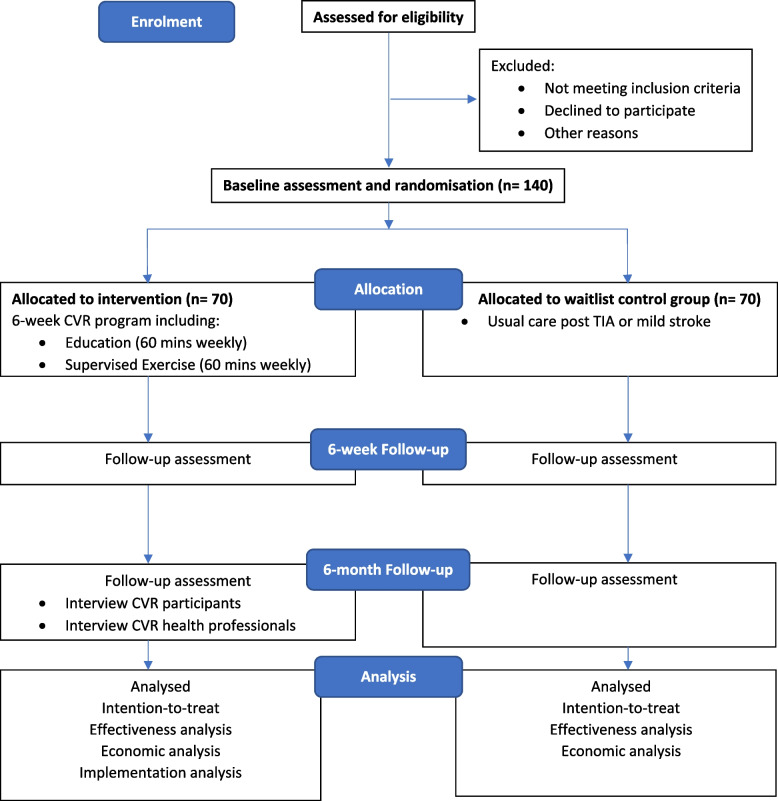
Flow of participants through the CRAMS trial**.** CVR, cardiovascular rehabilitation; TIA, transient ischaemic attack

The type 1 effectiveness-implementation hybrid study [36] will use a 2-arm single-centre assessor-blind randomised controlled trial design and will be conducted in Canberra, Australia, commencing in 2022 (Fig. 1). People with TIA and mild stroke will be randomised to a 6-week exercise-based Cardiovascular Rehabilitation (CVR) program or a usual care 6-month wait-list control group. Recruitment will be staggered over 18-months and the total study duration for each participant will be 6-months from commencing the study.

### Randomisation

An investigator not involved in recruitment or assessments will use a computer-generated random number sequence, with participants allocated in a 1:1 ratio. Group allocation will be concealed using sealed, consecutively numbered opaque envelopes. Following each participant’s baseline assessment, the next envelope in the sequence will be opened to reveal random allocation to one of two groups: the Cardiovascular Rehabilitation program or the 6-month wait-list control group.

### Participants

Participants will be recruited by health professionals from two hospitals (Canberra Health Services and Calvary Public Hospital Bruce) in-person or via mail using an existing TIA and stroke database, and from primary healthcare services within the Australian Capital Territory, Australia. All potential participants that express interest in participating in the study will be assessed by healthcare professionals and the research team to determine their eligibility. A research assistant will obtain written informed consent from all eligible participants on entry to the study (Supplementary File 1).

To be eligible, participants must be 18 + years old; have a medically documented TIA or mild non-disabling ischaemic stroke (National Institutes of Health Stroke Scale (NIHSS) score 0–4 [37]; modified Rankin Scale score ≤ 2 [38]) within the previous 12 months on discharge from the emergency department or the hospital ward, where applicable; and reside in the Australian Capital Territory, Australia. Participants will be excluded if they have an uncertain diagnosis; evidence of intracranial haemorrhage on a CT or MRI study; undergone recent (< 30 days) carotid endarterectomy; have evidence of a disabling stroke as measured by modified Rankin Scale score of ≥ 3; New York Heart Association class II-IV symptoms of heart failure; uncontrolled arrhythmias; severe chronic obstructive pulmonary disease; uncontrolled hypertension; symptomatic peripheral artery disease; unstable angina; uncontrolled diabetes; inadequate English language skills, including significant speech impairment, or cognitive skills; unable to perform a submaximal walking test or wear an accelerometer due to disability (e.g., confined to a wheelchair) or they have previously completed cardiac rehabilitation within the last 5 years.

### Cardiovascular Rehabilitation (CVR) program

The CVR program included in this study is typical of most contemporary phase II cardiac rehabilitation programs in Australia [14, 39]. The CVR program is multidisciplinary, time-limited (once a week, 6-weeks), conducted in groups, health service-based, and has educational and supervised exercise components (one hour education plus one hour exercise per week). Members of the multidisciplinary team include exercise physiologists, physiotherapists, dietitians, psychologists, pharmacists and supervised university health students. The intention of the CVR program is not to replace other treatment services that may be required after a mild stroke to address any deficits such as speech or motor impairments but rather provide a comprehensive program for CVD risk factor modification and management.

The main aim of the exercise sessions will be to increase exercise capacity by exercising large muscle groups at a moderate intensity [14]. The best predictor of an increased risk of death from any cause in men with cardiovascular disease is exercise capacity [40]. For every 1-MET increase in exercise, there is a 9% improvement in survival [40]. The exercise sessions will be conducted in the same way as a typical cardiac rehabilitation program as many cardiac rehabilitation participants have co-morbidities, including stroke [41]. All exercise sessions will be individually tailored for each of the participants, including aerobic and resistance training, and supervised by a physiotherapist and/or exercise physiologist.

Members of the multi-disciplinary team will conduct education sessions to increase the participant’s knowledge of their CVD condition and increase self-management skills for risk factor management. The education will be slightly modified compared to a typical cardiac rehabilitation program to include the anatomy and physiology of the brain, TIA and ischaemic stroke, and the management of stroke risk factors. The education sessions will cover: cardiac and brain anatomy and physiology; cardiac and stroke risk factors, modification and management of symptoms; how to deal with stress, anxiety and depression; cardiac and stroke medications; benefits of exercise and physical activity; and nutrition advice. People with heart disease will be recruited to this program to replicate a standard phase II cardiac rehabilitation program currently offered in Australia and to enable the integration of people with TIA, mild stroke, and heart disease in the same program. However, research outcomes will not be collected or analysed for this group as the evidence base for exercise-based cardiac rehabilitation for people with heart disease is well established [17].

### Usual care control group

Those randomised to the control group will participate in usual care following a TIA or mild stroke. In Australia, this typically consists of follow-up with a neurologist and little or no secondary prevention other than pharmacological management [42]. Control participants will be wait-listed for the CVR program and will commence the CVR program 6-months after they have completed their baseline measures.

### Outcome measures

All outcome measures will be collected at baseline, 6-weeks, and 6-months. Assessor blinding to group allocation will be used throughout the study. Research assistants will collect all outcome measures following training, using a standardised protocol and the same research grade equipment.

The primary outcome measure will be distance completed in the 6-min walk test and reported as metres (functional exercise capacity). Secondary outcome measures include daily minutes of accelerometer moderate-to-vigorous physical activity, body mass index, waist circumference, blood pressure, quality of life (AQoL-6D), anxiety and depression (HADS) and unplanned cardiovascular disease-related emergency department and hospital admissions. Qualitative data will be collected from participants and health professionals at the end of the intervention period, and cost analyses will be conducted. Demographic and clinical information will be collected using a questionnaire, including questions on sociodemographic variables (e.g., gender, age, education level) as well as clinical predictor variables (e.g., stroke-related medication, other medical conditions, smoking status). The research assistants will also record participant admission diagnosis and date of event or intervention details.

#### Functional exercise capacity

The 6-min walk test (6MWT) is a commonly used objective measure of functional exercise capacity in cardiac rehabilitation and stroke [43, 44]. The distance an individual is able to walk along a flat 25–30 m walkway over a 6-min period, with breaks as required, will be recorded. Only one trial will be administered. The test is a self-paced, submaximal test of exercise capacity, and has been found to have a moderate-to-high reliability and validity [44]. A change in distance of 34 m is considered a clinically meaningful change in people post stroke [43].

#### Physical activity levels

A triaxial commercial accelerometer (ActiGraph GT3X, Fort Walton Beach, FL) will be used to objectively assess physical activity. Accelerometers allow an objective measurement of quantity and intensity of movement and have been found to be reliable and valid [45]. Participants will be asked to wear the monitor on their right hip during a typical week while awake for 7-consecutive days. All data will be sampled and downloaded as raw data (30 Hz), converted to 15-s epochs (time interval), and then counts per minute (cpm) using the Actilife software [46, 47]. A ‘count’ is the unit of measure for activity for ActiGraph’s activity monitors [48]. Data will be screened, excluding data if: < 10 h per day wear time (non-wear defined as > 60 consecutive minutes where there is zero activity, with no allowance of epochs with counts above zero) and less than 4 days of valid data [46, 47, 49]. The Sasaki vector magnitude 3 cut-points will be used to determine time spent in MVPA (≥ 2690 cpm) [46, 47, 49, 50]. To measure sedentary behaviour, the vector magnitude cut-point will be used (< 150 cpm), categorizing light physical activity (LPA) as 150–2689 cpm [46, 47, 49, 51]. Estimating daily time spent in physical activity and sedentary behavior will be calculated by dividing the total time spent (minutes) in each threshold by the number of valid days.

#### Anthropometric characteristics and blood pressure

Height (m), weight (kg) and body mass index (BMI; kg/m^2^) will be recorded using a calibrated set of scales and a stadiometer. Waist circumference will be measured in centimetres using a stretch-resistant tape measure. The waist circumference will be recorded as the midpoint between the lower margin of the lowest palpable rib and the top of the iliac crest [52]. Blood pressure will be obtained using a sphygmomanometer (Welch-Allyn DS66) on the right arm of seated subjects. Waist circumference and blood pressure measurements will be repeated twice, and the average will be recorded.

#### Health-related quality of life

Health-related quality of life will be assessed using the Assessment of Quality of life (AQoL-6D). The AQoL-6D was developed in Australia and uses Australian importance weights [53]. The AQoL-6D is self-administered and consists of 20-items covering: independent living, mental health, coping, relationships, pain and sense. A multi-attribute utility score is then obtained, where a score of 100 reflects best health. The AQoL-6D is reliable and valid in community settings and can be used in economic analyses [54, 55].

#### Anxiety and depression

The Hospital Anxiety and Depression Scale (HADS) will be used for the assessment of anxiety and depression [56]. This questionnaire is a 14‐item self‐reporting questionnaire comprised of 4‐point Likert‐scaled items covering the occurrence of symptoms of anxiety (HADS‐A) and depression (HADS‐D) over the past 2 weeks. Each item on the questionnaire is scored from 0–3, so that a person can score between 0 (best outcome) and 21 (worst outcome) for either anxiety or depression. The minimally clinically important difference for patients with CVD is 1.7 [57].

#### Hospital admissions and emergency department visits

Participants will be surveyed at 6-weeks and 6-months to determine if they have had any unplanned hospital admissions or presentations to the emergency department during the study period. This information will then be verified, where possible, by hospital patient admission records to ascertain the Australian Refined Diagnosis Related Group (AR-DRG) and Urgent Related Group (URG) classification code assigned to the admission.

#### Qualitative perceptions of the CVR program

Qualitative data will be collected from the intervention participants. Semi-structured interviews will be conducted by a member of the research team (audio-recorded and professionally transcribed), investigating participants’ views on the acceptability of the intervention, including ease of attendance, satisfaction, and motivation. Interviews will be continued until the team is confident that no new ideas were being raised in the interviews.

At the completion of the CVR program health professionals involved in the program and referring to the CVR program will also be invited to take part in semi-structured interviews to investigate their views on the acceptability, value, and impact of the CVR program within health service delivery, as well as referral pathways. This data will be analysed and used to inform the integration of people with TIA and mild stroke into existing cardiac rehabilitation services, including the development of pathways to facilitate referrals to these services.

#### Feasibility

Data will be collected on participant recruitment rates, adherence to the program (number of exercise and education sessions attended), number of dropouts, and the number lost to follow-up. Any adverse events experienced during the program will also be recorded. If an adverse event occurs whilst the participant is taking part in the study, they will be managed immediately in line with university and health services policies and procedures. If there is an adverse event that is related to the intervention, the study coordinator will be made aware of group allocation and will discuss continuation of the participant’s involvement with the research team. Any adverse events will be reported to the ethics committee, advisory group and the participant’s medical team.

#### Costs

Direct intervention costs will be recorded prospectively. Healthcare resource use will include trial-intervention resource use (e.g., CVR clinician’s time) and non-trial healthcare use which may be impacted (e.g., hospital visits, where patients have consented to medical record access), costed using actual costs accrued where available or market rate estimates.

### Sample size

The sample size calculation for the 2-arm randomised control trial is based on an increase in 6MWT-distance as a result of aerobic exercise programs similar in design to cardiac rehabilitation exercise programs for survivors of stroke [58]. This systematic review and meta-analysis found an effect size of 0.41 (Hedges’ g corrected effect size) for 6MWT-distance. With a Cohen’s f = 0.205 (half Hedges’ g), three repeated measures (baseline, 6-weeks, and 6-months), a two-sided significance test at a 5% alpha level, a power of 80%, and assuming 0.5 as the correlation among repeated measures, the total sample size required is 128 (64 per arm), calculated using G*Power version 3.1.9.4. Assuming an 8% drop-out rate as commonly found in stroke research [59], the final sample size is 140, with 70 in each group.

### Data analyses

All participants who complete the baseline assessment and attend at least one CVR session will be included in the analysis. Descriptive statistics will be completed, and feasibility measures will be reported as percentages. Data will be analysed according to group assignment for participants with TIA or mild stroke following intention-to-treat principles. To analyse 6MWT-distance and all other secondary outcomes, methods for repeated measures analysis will be used. Linear (for continuous outcome data) and Generalised Linear (for Binary and Count outcome data) mixed-effects models for repeated measures will be used for between-within group comparisons and time-interactions analyses adjusting for demographic characteristics and other potential covariates, such as time since TIA or mild stroke. All analyses will be conducted using either SPSS or STATA. Any estimate will be reported with associated 95% confidence intervals. Significance level will be set at p < 0.05.

Implementation outcomes will be evaluated using both quantitative and qualitative methods. The Reach, Effectiveness, Adoption, Implementation, Maintenance (RE-AIM).

framework will be used to assess implementation outcomes such as characteristics of participants (reach), change in 6-min walk distance (effectiveness), number of referrers and characteristics of CVR program clinicians (adoption), fidelity and cost of the intervention (implementation) and drop-out rates (maintenance) [60]. A qualitative thematic analysis of semi-structured interviews from both participants and health professionals involved in the CVR program will also be undertaken. Two research team members will independently code and identify potential themes using an inductive thematic approach [61]. Emerging themes will be discussed until consensus is reached on final themes and categories. Deductive analyses will be mapped to the Consolidated Framework for Implementation Research (CFIR) [62] constructs to understand how the CVR program works and factors that influence implementation success or failure. The Intervention Characteristics and Inner Settings domains will be primarily considered as they are more relevant for this type 1 effectiveness-implementation hybrid trial.

The effect of the intervention on the rates of unplanned CVD-related hospital admissions and emergency department visits within the 6-months study period using a cost-effectiveness analysis will also be evaluated. Information on secondary measures such as health behaviour change (moderate-to-vigorous physical activity), BMI, waist circumference, quality of life (AQoL-6D), clinical and demographic information and the costs associated with the use and delivery of the CVR program will be used. A cost-utility analysis will be undertaken with a societal perspective using gained quality-adjusted life years (QALYs) as the measure of health effects. The impact on health-related quality of life can be measured using the AQoL-6D and the incremental cost-effectiveness ratios (ICERs) per QALYs will be calculated [63]. Information on the participants’ health service utilisation costs, based on the AR-DRG and URG classification codes will also be used. From these analyses, the additional cost per unit of health benefit gained over the lifetime (by using the CVR program compared to usual care) can be determined.

### Data management and dissemination of results

Participant paper and electronic files will be maintained in secure storage and on a password protected computer for a period of 5 years after completion of the study and will then be destroyed according to university protocols. Data entry screening will be conducted at the time of data entry to ensure no inconsistencies between paper-based and electronic data. Results will be disseminated through peer-reviewed academic journals, conferences, and webinars for health professionals. Results will be made available to participants on request.

## Discussion

Over 6-months we will assess the effectiveness and implementation of a CVR program for people with a TIA or mild stroke, providing recommendations on how to increase referrals and integrate this population into existing cardiac rehabilitation programs. There is limited evidence on the effectiveness of cardiac rehabilitation for people with TIA and stroke, whether programs are stroke-specific or integrated including people with heart disease [30–35]. Additionally, no studies appear to have investigated the cost-effectiveness or implementation determinants of such programs.

Cardiac rehabilitation is a well-established secondary prevention program that is currently available across Australia and internationally. Taking advantage of available cardiac rehabilitation programs may improve risk factors and decrease the risk of a heart attack or stroke in people with TIA and mild stroke. A modelling study in 2007 has shown that non-pharmacological strategies after stroke could result in an 80% reduction in further vascular events [64]. There is also evidence that people who have had a TIA or mild stroke want to understand their diagnosis and residual impairments, their risk of recurrent events and they want tailored advice and support on lifestyle modifications to improve their health and quality of life [42]. Currently, health care is medically focused, with medical investigations and stroke prevention medication prioritised in this population, and follow-up care is variable and fragmented [42].

There are some limitations to this trial. Multiple assessors of outcome measures will be used to maintain assessor blinding at all time points. Identical calibrated equipment and standard outcome measurement training will be provided to all assessors to account for this. Other possible limitations include low recruitment rates and potential for loss of participants to follow-up [65]. According to the TIA and stroke database maintained by the hospitals involved in the trial and assuming a 34% recruitment rate as commonly found in stroke research [59], an 18-month recruitment period should be sufficient to recruit the required sample size, with low dropout rates commonly reported in this population [59].

One third of all heart attacks and strokes are repeat events. Cardiac rehabilitation reduces the risk of heart attacks and strokes but it is not routinely offered to people who have had a TIA or mild stroke. If positive effectiveness and implementation outcomes are found, an integrated CVR program will be promoted, potentially improving health outcomes and quality of life of people who have had a TIA or mild stroke and reducing their risk of recurrent CVD events.

## Supplementary Information

**Additional file 1.** CRAMS Trial Participant Information Form.

## Data Availability

Not applicable.

## Abbreviations

6MWT: 6-Minute walk test
AQol-6D: Assessment of Quality of life
AR-DRG: Australian Refined Diagnosis Related Groups
BMI: Body mass index
CFIR: Consolidated Framework for Implementation Research
CVD: Cardiovascular disease
CVR: Cardiovascular rehabilitation
HADS: Hospital Anxiety and Depression Scale
ICERs: Incremental cost-effectiveness ratios
NIHSS: National Institutes of Health Stroke Scale
QALYS: Quality-adjusted life years
RE-AIM: Reach, Effectiveness, Adoption, Implementation, Maintenance
TIA: Transient ischaemic stroke
URG: Urgency Related Groups
