# Supplementary material for: Cardiovascular Rehabilitation for transient ischaemic Attack and Mild Stroke: the CRAMS effectiveness-implementation hybrid study protocol
Source: BMC Health Serv Res. 2022 Nov 22;22:1391. doi: 10.1186/s12913-022-08797-3 (PMC9682670; doi:10.1186/s12913-022-08797-3)
Supplement: Supplementary file 1 — Additional file 1. CRAMS Trial Participant Information Form. [file 12913_2022_8797_MOESM1_ESM.docx]

## Participant Information Form

SUPPLEMENTARY FILE 1

**Cardiac Rehabilitation for Transient Ischaemic Attack and Mild-Stroke: the CRAMS trial**

**Research Team**
A/Prof Nicole Freene (University of Canberra, UC), A/Prof Elisabeth Preston (UC), Allyson Flynn (UC), Shahla Cowans (Canberra Hospital), Hannah Wallett (UC), A/Prof Theo Niyonsenga (UC), A/Prof Itismita Mohanty (UC), Prof Christian Lueck (Canberra Hospital), Prof Rachel Davey (UC), Inga Eveston (Research Assistant)

**Project Aim**

Stroke, transient ischaemic attack (TIA) and heart disease, all forms of cardiovascular disease, share common risk factors, such as not enough physical activity, smoking, high blood pressure and poor diet. One in three strokes and heart attacks in Australia are repeat events, and guidelines recommend that every person with cardiovascular disease should be referred to a secondary prevention program, such as cardiac rehabilitation, to prevent these repeat events.

Cardiac rehabilitation is a comprehensive secondary prevention program which addresses risk factors for cardiovascular disease using exercise and education. Our previous research has found that despite the proposed suitability of cardiac rehabilitation, people who have suffered a TIA or mild-stroke are not attending cardiac rehabilitation programs in Australia.

The aim of this research is to investigate the effectiveness of a novel 6-week exercise-based cardiac rehabilitation program for people who have had a TIA or mild-stroke (Cardiovascular rehabilitation, CVR) at the University of Canberra (UC) Health Clinics compared to usual care.

**Benefits of the Project**

If this study has positive outcomes, the cardiovascular rehabilitation program will improve health outcomes and quality-of-life of Canberrans who have had a TIA or mild-stroke. Findings from this study will guide future research, policy and practice, and ultimately reduce repeat heart attacks and strokes for this population.

**Participant Involvement**

To participate in this research you will be asked to agree to being randomly allocated to the intervention group (CVR program) or usual care. If participants are randomly allocated to the intervention group they will commence the CVR program as soon as possible. Participants in the usual care group will be placed on a 6-month waiting-list and will be offered the CVR program after 6-months, although they will not be required to attend the CVR program at this time if they do not wish to.

The CVR program will be run once a week, for 2-hours at a time, for 6-weeks at the UC Health Clinics in Bruce. The CVR program is multidisciplinary, conducted in groups and has educational and supervised exercise components (one hour education plus one hour exercise). Members of the multidisciplinary team include exercise physiologists, physiotherapists, dietitians, psychologists, pharmacists and supervised UC health students. All exercise sessions will be individually tailored for each of the participants and supervised by a physiotherapist and/or exercise physiologist. Members of the multi-disciplinary team will conduct education sessions to increase the participant’s knowledge of their condition and increase self-management skills for risk factor management. The education sessions will cover: cardiac and brain anatomy and physiology; cardiac and stroke risk factors, modification and management of symptoms; how to deal with stress, anxiety and depression; cardiac and stroke medications; benefits of exercise and physical activity; and nutrition advice. People with heart disease will also be attending the CVR program. Intervention participants will be encouraged to attend all 6 sessions during the 6-week CVR program.

Assessment measures will be collected from all participants at baseline, 6-weeks and 6-months. All assessments for all participants (intervention and usual care) will be conducted at the UC Health Clinics and will take approximately 30-minutes to complete. Assessment measures will involve:

- a sub-maximal walking test to assess your exercise capacity (6-minute walk test)
- wearing a physical activity monitor (accelerometer) on your waist, while awake, over 7-consecutive days, recording information about your physical activity patterns
- completion of a quality-of-life questionnaire and a questionnaire to assess anxiety and depression (approximately 5-minutes to complete)
- blood pressure measured with via a sphygmomanometer
- waist and hip measurements using a tape measure
- height and weight measures
- examination of your medical records for any unplanned hospital admissions or presentations to the emergency department during the 6-month study period
- some of you in the intervention group will be asked to take part in an interview to investigate your views on the acceptability of the CVR program, including ease of attendance, satisfaction and motivation (approximately 30-minutes to complete).

Participation in this research is completely voluntary and you may, without any penalty, decline to take part or withdraw at any time without providing an explanation or refuse to answer a question. It is completely up to you whether or not you participate. If you decide not to participate, it will not affect the treatment you receive now or in the future. Whatever your decision, it will not affect your relationship with the staff caring for you.

**Confidentiality**

Only the researchers will have access to the individual information provided by participants. Privacy and confidentiality will be assured at all times. The research outcomes may be presented at conferences and written up for publication. However, in all these publications, the privacy and confidentiality of individuals will be protected.

**Anonymity**

All reports and publications of the research will contain no information that can identify any individual and all information will be kept in the strictest confidence.

**Data Storage**

The information collected will be stored securely on a password protected computer throughout the project and in a locked filing cabinet (paper copies) at the University of Canberra. Data will then be stored at the University of Canberra for the required five year period after which it will be destroyed according to university protocols.

**Ethics Committee Clearance**

The project has been approved by the Human Research Ethics Committees of the University of Canberra (HREC – 9351) and Calvary Public Hospital Bruce (40-2021).

**Queries and Concerns**

As part of the assessment, you will be required to complete a walking test. The less than maximal effort walking test may make you feel short of breath, dizzy, nauseous, and may cause leg or chest pain. The test may be terminated by you at any stage or by the assessor if criteria for stopping the exercise test are met, that is, chest pain, increased shortness of breath, leg cramps, dizziness, confusion, pale appearance, nausea, cold and clammy skin, failure of your heart rate to increase with increasing exercise levels and severe fatigue.

If you suffer any injuries or complications as a result of this study, you should contact the study team as soon as possible, who will assist you in arranging appropriate medical treatment, or visit your GP and/or hospital emergency department as required.

If the questions asked in this study result in feelings of discomfort or distress, please contact your General Practitioner or LifeLine 13 11 14 to discuss further.

Queries or concerns regarding the research can be directed to A/Prof Nicole Freene, phone 02 6201 5550 or email [nicole.freene@canberra.edu.au](mailto:nicole.freene@canberra.edu.au)

If you have any complaints or reservations about the ethical conduct of this research, you may contact the University of Canberra’s Research Ethics & Integrity Unit team via telephone 02 6206 3916 or email [humanethicscommittee@canberra.edu.au](mailto:humanethicscommittee@canberra.edu.au) or [researchethicsandintegrity@canberra.edu.au](mailto:researchethicsandintegrity@canberra.edu.au)

If you would like some guidance on the questions you could ask about your participation please refer to the Participants’ Guide located at <http://www.canberra.edu.au/ucresearch/attachments/pdf/a-m/Agreeing-to-participate-in-research.pdf>

# Consent Form

**Project Title**

**Cardiac Rehabilitation for Transient Ischaemic Attack and Mild-Stroke: the CRAMS trial**

**Consent Statement**

I have read and understood the information about the research. I am not aware of any condition that would prevent my participation, and I agree to participate in this project. I have had the opportunity to ask questions about my participation in the research. All the questions I have asked, have been answered to my satisfaction.

Please indicate whether you agree to participate in each of the following parts of the research (please indicate which parts you agree to by putting a cross in the relevant box):

□ Randomised to the intervention or usual care (6-month waiting-list) group.

□ Complete a sub-maximal walking test to assess exercise capacity.

□ Wear a physical activity monitor on your waist, while awake, over 7-consecutive days.

□ Complete 2 brief questionnaires on quality-of-life and anxiety & depression.

□ Have height, weight, waist & hip circumference and blood pressure measured.

□ Examination of your medical records for any unplanned hospital admissions or presentations to the emergency department during the 6-month study period.

□ Participate in an interview with the researcher which will be audio-recorded.

Name……………………………………………………………………….……………………........…

Signature………….........................................................………………………………

Date ………………………………………………………….

A summary of the research report can be forwarded to you when published. If you would like to receive a copy of the report, please include your mailing (or email) address below.

Address/Email…………………………………………………………………………………………

……………………………………………………………………………………………….....……………
